# Metformin Impacts Human Syncytiotrophoblast Mitochondrial Function from Pregnancies Complicated by Obesity and Gestational Diabetes Mellitus in a Sexually Dimorphic Manner

**DOI:** 10.3390/antiox12030719

**Published:** 2023-03-14

**Authors:** Jessica F. Hebert, Leslie Myatt

**Affiliations:** Department of Obstetrics and Gynecology, Oregon Health & Science University; Portland, OR 97239, USA

**Keywords:** placenta, metformin, mitochondrial dysfunction, sexual dimorphism

## Abstract

Maternal obesity and gestational diabetes mellitus (GDM) are associated with placental dysfunction, small for gestational age (SGA) offspring, and programming of adult-onset disease. We examine how metformin, commonly used to treat type A2 GDM, affects placental metabolism as well as mitochondrial content and function. Syncytiotrophoblasts (STBs) were prepared from placentas of male and female fetuses collected at term cesarean section from lean (pre-pregnancy BMI < 25), obese (BMI > 30), and obese A2GDM women. Metformin treatment (0.001–10 mM) of STB caused no change in non-mitochondrial respiration but significant concentration-dependent (1 and 10 mM) decreases in basal, maximal, and ATP-linked respiration and spare capacity. Respiration linked to proton leak was significantly increased in STB of male A2GDM placentas at low metformin concentrations. Metformin concentrations ≥1 mM increased glycolysis in STB from placentas from lean women, but only improved glycolytic capacity in female STB. Whereas metformin had little effect on superoxide generation from male STB of any group, it gave a concentration-dependent decrease in superoxide generation from female STB of lean and obese women. Fewer mitochondria were observed in STB from obese women and male STB from lean women with increasing metformin concentration. Metformin affects STB mitochondrial function in a sexually dimorphic manner but at concentrations above those reported in maternal circulation (approximately 0.01 mM) in women treated with metformin for GDM.

## 1. Introduction

In the United States, 39.7% of women of reproductive age are obese (BMI > 30) [1], a significant health concern linked to an increased risk of hypertension and diabetes [2] and a greater risk for pregnancy complications. Obese women are twice as likely to develop preeclampsia and three times more likely to have gestational hypertension or gestational diabetes mellitus (GDM) [3,4]. GDM affects 7% of pregnancies and is associated with increased circulating maternal glucose and insulin resistance, resulting in fetal hyperglycemia and increased fetal insulin secretion. Consequently, the risk of pregnancy complications and postnatal consequences for mother and offspring, including later onset of diabetes, obesity, and cardiovascular disease in both, is also elevated [5,6,7]. Babies from mothers with obesity and GDM are more likely to be born small for gestational age (SGA), which is further linked to the developmental programming of adult-onset disease [8,9].

Not all GDM pregnancies are complicated by obesity, nor do all obese women develop GDM. However, as obesity increases the risk of GDM and other health issues during gestation, studying these risks and the effects of current therapeutic interventions is essential in obstetric research, especially concerning their effects on placental function. The placenta is the primary regulator of pregnancy, producing hormones to direct maternal metabolism to mobilize substrates to support fetal growth, modulating nutrient, gas, and waste exchange, while also serving as a physical and immunological barrier between mother and fetus [10]. The placenta, particularly the trophoblast cells, has substantial metabolic activity and requires energy to sustain these critical functions. The two-tiered villous trophoblast layer is responsible for oxygen and nutrient uptake from maternal circulation and delivery to the developing fetus. Mononucleated villous cytotrophoblasts (CTB) form the underlying proliferative layer and fuse to form the outer layer of multinucleated syncytiotrophoblasts (STB), where contact with the maternal circulation and nutrient uptake takes place. In addition to nutrient transport, STB are responsible for the synthesis and secretion of substantial amounts of peptide and steroid hormones [11]. Hence, the placenta consumes a large proportion of substrates reaching it, with oxygen and glucose consumption six times that of the fetus per unit weight [12] while being only one sixth of the size. Measuring trophoblast mitochondrial respiration and glycolytic activity (the consumption of the primary fuels oxygen and glucose) is an effective index of placental metabolic activity. We have previously used the Seahorse extracellular flux analyzer (Agilent) to assess respiration in primary STB cultures and showed significantly reduced mitochondrial respiration with increasing maternal adiposity [13], with an even further reduction in STB from obese women with medication-controlled type A2 gestational diabetes (A2GDM) [14]. In addition, human STB showed a lack of fuel flexibility (the ability to switch between glucose, fatty acid, and glutamine as substrates for mitochondrial respiration) with increasing maternal adiposity and A2GDM, and with male fetal sex [5]. Male fetuses are well known to be at higher risk for adverse outcomes, including stillbirth and developmental programming of adult-onset disease. This is particularly true in pregnancies complicated by obesity and GDM [15,16,17], which are further associated with differences in gene expression in male versus female placentas [18,19,20], antioxidant defenses [21], mitochondrial respiration [22], and mitochondrial biogenesis [23].

**Figure 1:**
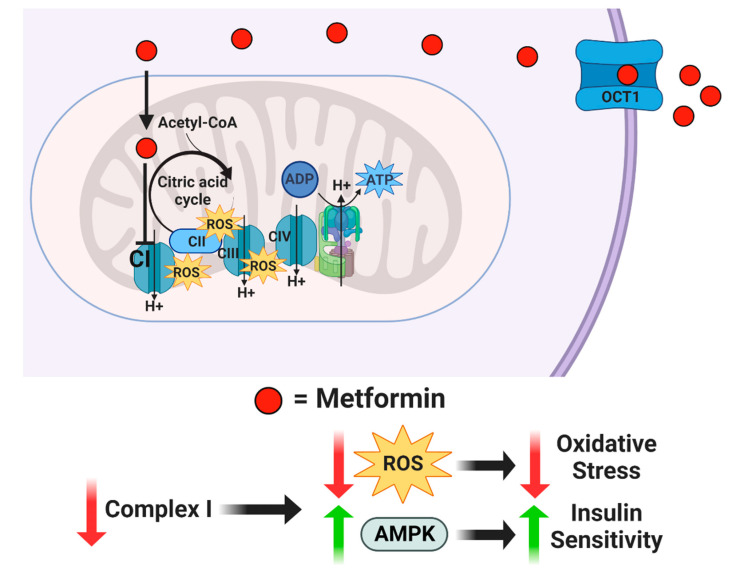
Proposed mechanism of action for metformin. Metformin enters the cell through organic cation transporter 1 (OCT1) and is transferred into the mitochondria by its positive net charge. Once inside the mitochondrial matrix, metformin inhibits Complex I, which reduces reactive oxygen species while increasing AMPK.

For more than forty years, drugs, such as insulin, glyburide, and metformin, have been used to attempt to improve A2GDM outcomes [24]. The mechanism of action of metformin is unclear, but it is suspected to inhibit mitochondrial complex I, which reduces mitochondrial respiration and ATP production, concurrently inhibiting the synthesis of reactive oxygen species (ROS) and generation of oxidative stress [25,26]. In addition, metformin appears to increase AMPK and insulin sensitivity, which enhances glucose transport [27] (Figure 1). Although insulin and glyburide cross the placenta minimally, recent studies indicate that metformin concentrations in fetal cord blood range from half to nearly equal to that in maternal plasma [28,29]. Tarry-Adkins et al. found that while fetal plasma metformin concentration is roughly one-and-a-half times that in maternal plasma, metformin concentrations in both maternal and fetal plasma correlated to placental metformin concentrations [30]. When metformin is used to treat GDM, the concentration in maternal circulation is approximately 0.01 mM, but with putatively much higher mitochondrial accumulation [31,32,33,34,35]. Previous studies exploring metformin’s mechanisms of action employed much higher concentrations in human cell cultures (2 mM) [36], non-human cell cultures (0.1 mM) [25], and non-human-derived whole and partial mitochondria (100 mM) [26]. Therefore, the full range of metformin effects at a physiologically relevant concentration on syncytiotrophoblast metabolic function are unknown.

As we have previously shown that maternal obesity and A2GDM are associated with reduced trophoblast respiration in a sexually dimorphic manner [13,14,37], given the potential ameliorative effect of metformin on mitochondrial activity, here, we investigate the effect of a range of metformin concentrations on mitochondrial respiration, glycolysis, and mitochondrial activity, as assessed by membrane potential and generation of ROS in STB from lean, obese, and type A2GDM women with either a male or a female fetus.

## 2. Materials and Methods

### 2.1. Ethical Approval and Study Participants

Placentas were collected from the Labor and Delivery Unit at Oregon Health & Science University Hospital for the OHSU Placenta Repository per a protocol approved by the university Institutional Review Board (Study ID: 00016328) with informed consent from the patients. All tissues and clinical data were deidentified before researchers were given access.

Placentas were collected from three groups of women with either a male or female fetus: lean (pre-pregnancy BMI 18.5–25), obese (pre-pregnancy BMI 30–45), and A2GDM (GDM controlled by insulin, matched for BMI with obese women). Patient, fetal, and placental characteristics are summarized in Table 1. Patients had no other pregnancy complications (e.g., preeclampsia, asthma, IUGR) and no reported co-morbidities, such as smoking or drug use.

### 2.2. Tissue Collection, Trophoblast Isolation, and Cell Culture

Placentas were collected at term by cesarean section in the absence of labor immediately following delivery of the neonate. Villous tissue (∼60 g, in roughly 2.5 cm^2^ pieces) was collected from several random sites in the placenta, and primary cytotrophoblasts were isolated using a well-validated protocol [37,38,39,40]. Briefly, tissue was rinsed in PBS and trophoblasts scraped away from the chorionic plate and blood vessels before being digested three times in HEPES Buffered Salt Solution containing Trypsin and DNase at 37 °C. Cells collected by centrifugation underwent Percoll gradient purification, were counted, and kept frozen in liquid nitrogen in FBS/DMSO freezing media until culture. Syncytiotrophoblasts were created by culturing cytotrophoblasts in 96-well cell culture plates at a concentration of 100,000 cells/well for 72 h in Iscove’s Modified Dulbecco’s Medium (IMDM supplemented with 10% FBS and 1% penicillin/streptomycin). In vitro, cytotrophoblasts spontaneously fuse and differentiate to form multinucleated STB [13,40]. The identity and purity of syncytiotrophoblasts were confirmed, by light microscopy and immunofluorescence with cytokeratin 7 and DAPI [38,39,41] and the production of hCG upon syncytialization. Following syncytialization, STB were treated for an additional 24 h with 0–10 mM metformin in IMDM before Seahorse assays.

### 2.3. Mitochondrial Respiration

**Figure 2:**
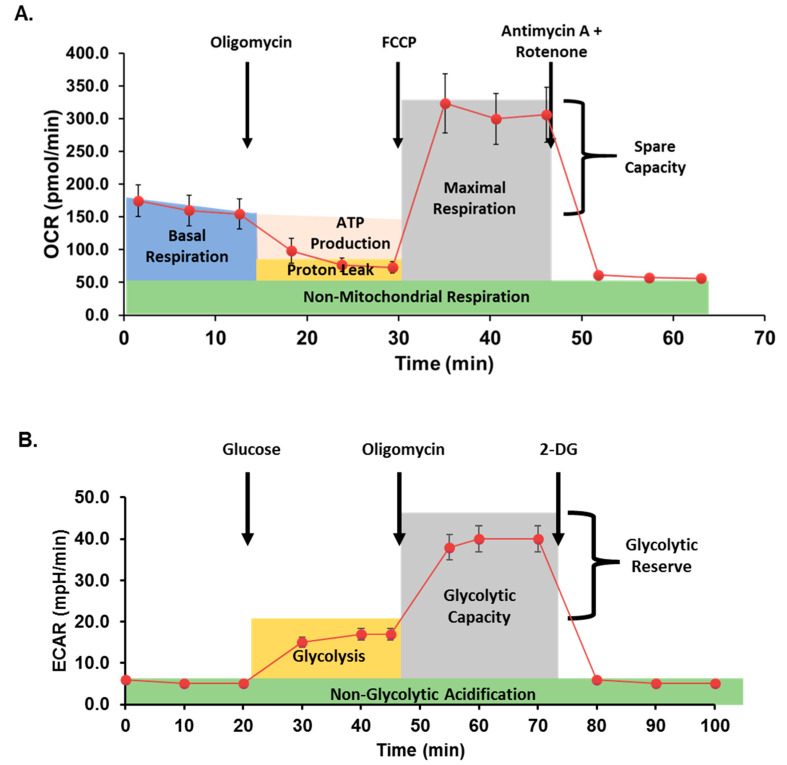
Seahorse assay method schematic. (**A**) In mitochondrial stress assays, oxygen consumption rate (OCR) is measured before and after additions of oligomycin (an inhibitor of ATP synthase), carbonyl cyanide p-trifluoromethoxy-phenylhydrazone (FCCP; proton gradient uncoupler), and antimycin A + rotenone (inhibitors of mitochondrial complex I and III, respectively) to quantify mitochondrial respiration. (**B**) In glycolytic stress assays, extracellular acidification rate (ECAR) is measured after inducing glycolysis, inhibiting ATP synthase, and finally inhibiting glycolysis.

Mitochondrial respiration was assessed in cells with or without metformin treatment using the Seahorse Bioscience XFe96 analyzer and the Mito Stress Test Kit (both Agilent) as previously described [5,38]. Data are expressed as the rate of oxygen consumption (OCR) in pmoles/min under basal conditions and following sequential injection of oligomycin (10 μM), carbonyl cyanide p-trifluoromethoxy-phenylhydrazone (FCCP; 10 μM), and rotenone/antimycin A (10 μM) to measure ATP-coupled respiration, maximal respiration, spare capacity, and non-mitochondrial respiration. Data were normalized to total cellular DNA per well as quantified using the Quant-iT PicoGreen dsDNA Assay (ThermoFisher). A typical curve obtained from the Seahorse analyzer during a Mito Stress assay is illustrated in Figure 2A.

### 2.4. Glycolysis

Glycolysis was assessed by Seahorse XF Cell Glycolysis Stress Test (Agilent), as previously described [42]. Briefly, following treatment with or without metformin, cells were serum-starved for one hour in XF Base Medium supplemented with 4 mM L-glutamine. The sequential glycolytic stress test injections contained glucose (100 mM), oligomycin (10 μM), and 2-Deoxyglucose (2DG; 500 mM; Sigma). Extracellular acidification rate (ECAR, mpH/min) was recorded for three cycles following each timed injection and normalized to total cellular DNA per well as quantified above. Figure 2B demonstrates a typical glycolysis stress analysis after treatment with glucose and inhibitors.

### 2.5. Mitochondrial Superoxide Production

MitoSOX Red (Invitrogen) rapidly targets mitochondria in live cells and is oxidized by superoxide to produce red fluorescence. STB were stained with MitoSOX Red (5 μM) in HBSS at 37 °C and 5% CO_2_ for 30 min. Cells were washed with warm HBSS three times. Fluorescence (510 nm excitation, 580 emission) was measured using a BioTek Synergy H1 hybrid plate reader (BioTek). Data were normalized to total cellular DNA per well, as measured with Quant-iT PicoGreen dsDNA Assay (ThermoFisher).

### 2.6. Quantification of Active Mitochondria

MitoTracker Deep Red (Invitrogen) passively diffuses across the plasma membrane and accumulates in active mitochondria dependent on mitochondrial potential. Following the various treatments, STB were stained with MitoTracker Deep Red (200 nM) in Hank’s Buffered Saline Solution (HBSS) in an incubator at 37 °C and 5% CO_2_ for 30 min. Cells were washed with warm HBSS three times. As above, fluorescence (644 nm excitation, 665 nm emission) was measured using a plate reader. Data were normalized to total cellular DNA per well as quantified above.

### 2.7. Statistical Analysis

Data for each parameter measured in the Mito and Glyco stress tests at each concentration of metformin were normalized to values obtained in the same batch of cells with no treatment. Data are reported as mean +/− standard deviation. Comparisons between groups were performed by two-way ANOVA with Tukey post hoc test for multiple comparisons using GraphPad Prism. *p* < 0.05 was considered significant for these analyses.

## 3. Results

### 3.1. Clinical Characteristics

There were no significant differences in maternal age, gestational age at delivery, fetal weight, or placental weight between lean, obese, and A2GDM groups. By experimental design, women with A2GDM were BMI matched to obese women. Both groups had significantly higher BMI than lean women. The fetal/placental weight ratio of males from obese and A2GDM women was significantly lower than lean women (*p* < 0.05). Females from obese women had a smaller fetal/placental weight ratio compared to females from A2GDM women and lean women (*p* < 0.05). These findings are summarized in Table 1.

### 3.2. The Effect of Metformin on Mitochondrial Respiration

**Figure 3:**
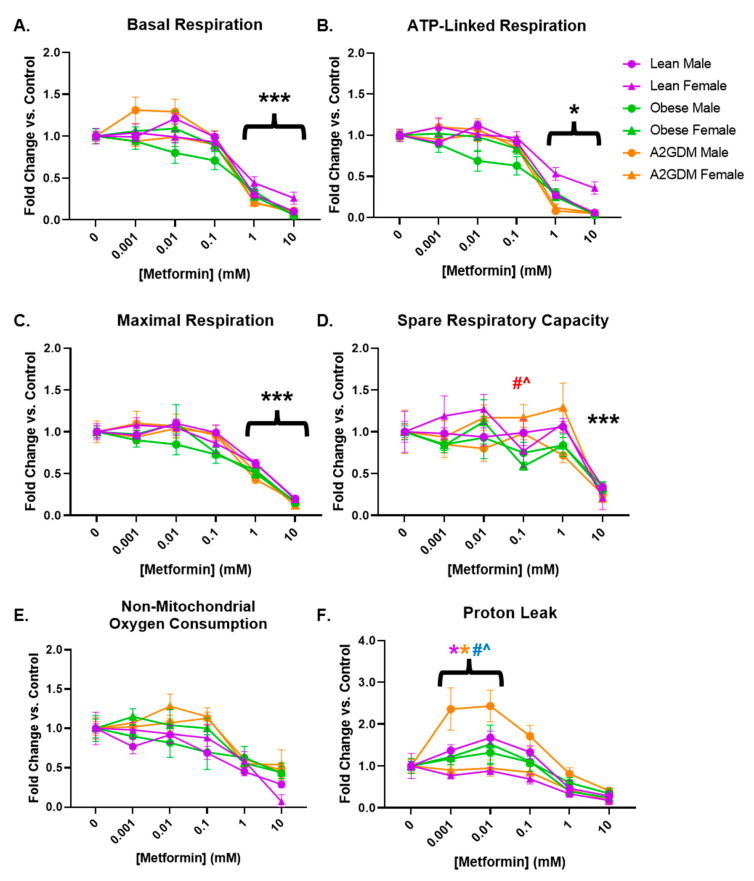
Mitochondrial respiration parameters following metformin treatment: (**A**) basal respiration, (**B**) ATP-linked respiration, (**C**) maximal respiration, (**D**) spare respiratory capacity, (**E**) non-mitochondrial oxygen consumption, and (**F**) oxygen consumption related to proton leak in syncytiotrophoblast of lean, obese and A2GDM women with either a male or female fetus. Values, normalized to corresponding no treatment control, expressed as means ± SD. Statistical assessment was performed using 2-way ANOVA with Tukey post hoc. Level of significance: One symbol: *p* < 0.05. Two same symbol: *p* < 0.01. Three same symbol: *p* < 0.001. *: Significant difference in all groups vs. control (no metformin). Difference at indicated metformin concentrations: Between sexes within group: Lean *, Obese *, A2GDM *. Lean vs. Obese **Males** † and Females †. Lean vs. A2GDM Males # and Females #. Obese vs. A2GDM Males ^ and Females ^.

Basal respiration, a measure of oxygen consumption in resting cells, was decreased in a concentration-dependent manner in STB after treatment with increasing amounts of metformin (*p* < 0.001 from 1 mM onward) in both male and female cells from all maternal conditions (Figure 3A). Although there was a decrease in basal respiration in STB from male placentas of obese mothers at lower concentrations of metformin, there was no significant difference between them and the trophoblast of male placentas of other conditions, nor between them and the trophoblast of obese females.

Following the inhibition of ATP synthase with oligomycin (Figure 2), the resulting decrease in oxygen consumption reveals the proportion of basal respiration used to generate ATP. STB from all groups of patients, regardless of maternal condition or fetal sex, had decreased ATP-linked respiration after treatment with 1–10 mM metformin (*p* < 0.05 from 1 mM onward, Figure 3B).

Metformin had a similar effect on maximal respiration, measured after administration of FCCP, which permeabilizes the mitochondrial membrane and permits the free flow of protons as ATP drivers. Regardless of maternal condition or fetal sex, STB of all groups showed a concentration-dependent decrease in maximal respiration following at least 1 mM metformin treatment (*p* < 0.001 from 1 mM onward, Figure 3C). Spare capacity, i.e., the difference between basal respiration and maximal respiration, representing the cell’s ability to increase respiration in response to stress, did not decrease significantly until treatment with 10 mM metformin in all patient groups (Figure 3D) due to the more gradual decrease in maximal respiration (Figure 3C) versus that of basal respiration (Figure 3A) with increasing concentrations of metformin. However, the spare capacity of STB of females from A2GDM women was increased vs. corresponding female STB of both lean (52%, *p* < 0.01) and obese (98%, *p* < 0.001) women at 0.1 mM metformin (Figure 3D).

No significant change was observed with increasing amounts of metformin in non-mitochondrial respiration, the proportion of oxygen consumption that occurs outside of oxidative phosphorylation (Figure 3E). However, the difference between non-mitochondrial respiration and oxygen consumption related to ATP generation indicates oxygen consumption linked to proton leak from the electron transport chain. OCR related to proton leak in STB from obese and GDM women increased at low metformin concentrations compared to STB from lean women (Figure 3F). Indeed, male STB from A2GDM women had significantly increased OCR related to proton leak compared to females from A2GDM women (150%, *p* < 0.01), as well as from males from obese women (126%, *p* = 0.05) and lean women (106%, *p* = 0.003) at 0.01 mM metformin. Males from lean women also had increased OCR related to proton leak compared to females from lean women after treatment with 0.001 to 0.1 mM metformin (57%, *p* < 0.05 at 0.01 mM metformin).

### 3.3. Glyco Stress Assays

**Figure 4:**
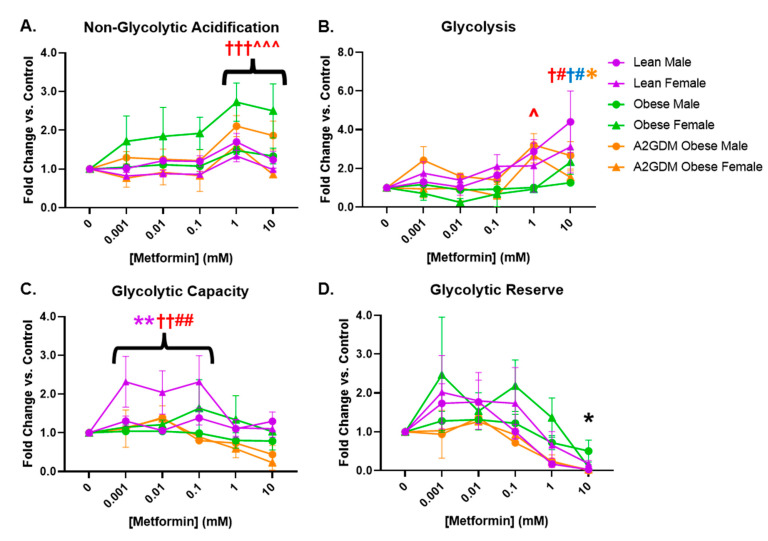
Glycolytic stress in syncytiotrophoblasts following metformin treatment as measured by (**A**) acidification not related to glycolysis, (**B**) glycolysis, (**C**) glycolytic capacity, and (**D**) glycolytic reserve. Values, normalized to no treatment controls, expressed as means ± SD. Statistical assessment was performed using 2-way ANOVA with Tukey post hoc. Level of significance: One symbol: *p* < 0.05. Two same symbol: *p* < 0.01. Three same symbol: *p* < 0.001. Difference at indicated metformin concentrations: Between sexes within group: Lean *, Obese *, A2GDM *. Lean vs. Obese **Males †** and **Females** **†**. Lean vs. A2GDM **Males****#** and **Females #**. Obese vs. A2GDM **Males ^** and **Females ^**.

Similar to measuring mitochondrial respiratory stress via oxygen consumption, glycolysis can be measured by the change in acidification of cell culture medium, i.e., the extracellular acidification rate (ECAR). Female STB from obese women and male STB from A2GDM women exhibited elevated non-glycolytic acidification (caused by processes other than glycolysis) with 1- and 10-mM metformin compared to untreated controls (*p* < 0.01) and to other maternal and fetal sex-matched groups (*p* < 0.05, Figure 4A). Metformin significantly increased glycolysis only in male (1 and 10 mM, *p* < 0.05, *p* < 0.01) and female STB (10 mM, *p* < 0.05) from lean but not obese or A2GDM women (Figure 4B). Only female STB from lean women had significantly increased glycolytic capacity (the ability to acutely increase conversion of glucose to pyruvate or lactate) at 0.001–0.1 mM metformin (*p* < 0.01). No significant differences in glycolytic reserve (the ability to respond to an increase in demand) were found between groups with increasing metformin concentrations (Figure 4C,D).

### 3.4. Determination of Superoxide Release

**Figure 5:**
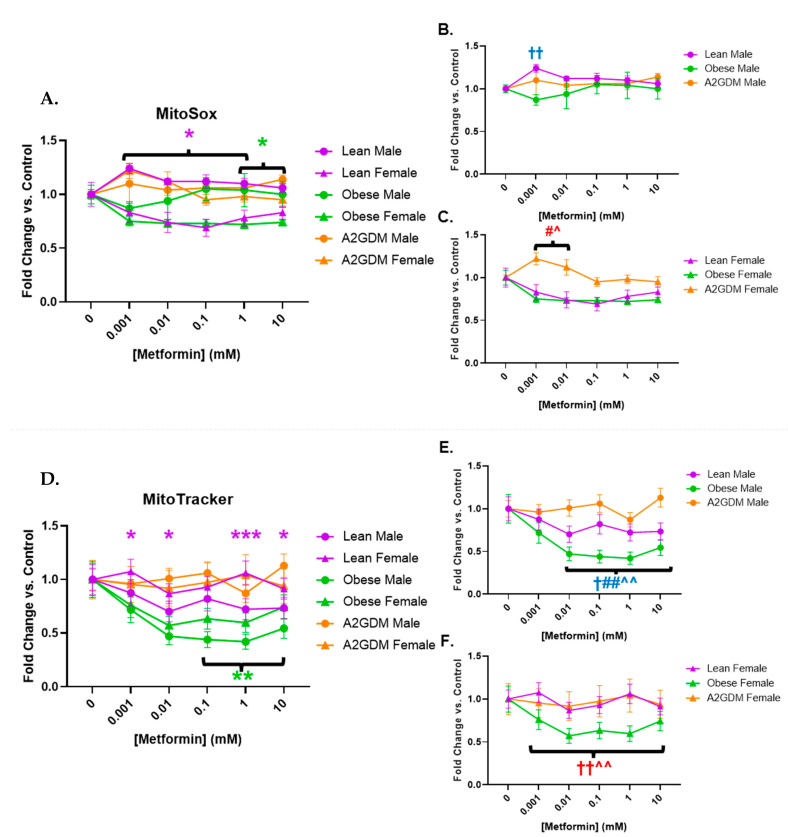
Changes in mitochondrial superoxide production and active mitochondria in response to metformin. (**A**) MitoSox Red measurement of superoxide production with all groups combined, (**B**) males only, and (**C**) females only. (**D**) Mitochondrial activity quantification measured by fluorescence using MitoTracker with all groups combined, (**E**) males only, and (**F**) females only. Values, normalized to no treatment controls, expressed as means ± SD. Statistical assessment was performed using 2-way ANOVA with Tukey post hoc. Difference at indicated metformin concentrations: Between sexes within group: Lean *, Obese *, A2GDM *. Lean vs. Obese **Males †** and **Females** **†**. Lean vs. A2GDM **Males****#** and **Females #**. Obese vs. A2GDM **Males ^** and **Females ^**.

Metformin treatment appeared to affect superoxide generation in a sexually dimorphic manner, as well as in relation to pathology. Female STB treated with increasing concentrations of metformin showed a significant reduction in superoxide generation when from lean women at 0.001–1 mM, and from obese women at 1–10 mM (both *p* < 0.05), vs. STB from corresponding male groups (Figure 5A). Increasing metformin concentrations had little effect on superoxide generation from STB of male placentas although superoxide generation in male STB of lean women was significantly greater at 0.001 mM metformin vs. male STB of obese or A2GDM women (*p* < 0.01, Figure 5B). Superoxide generation in female STB of the A2GDM group was resistant to the effect of increasing metformin, being significantly greater at 0.001–0.01 mM vs. lean or obese, but then fell with metformin concentrations above 0.1 mM (Figure 5C).

### 3.5. Quantification of Active Mitochondria

Mitochondria activated by membrane potential decreased in male compared to female STB of lean women with increasing metformin concentrations. Metformin concentrations above 0.1 mM caused a reduction in mitochondrial activity in STB from obese women vs. no treatment with a more pronounced effect in males than females (*p* < 0.01) (Figure 5D). Any metformin treatment caused a reduction in mitochondrial activity in male STB from lean and obese women (*p* < 0.05 for lean, *p* < 0.001 for obese), a response that was not shared by male STB from A2GDM women (Figure 5E). Metformin treatment also resulted in significantly less mitochondrial activity in female trophoblast from obese women compared to lean or A2GDM women (*p* < 0.01 (Figure 5F)).

## 4. Discussion

We investigated the effect of a commonly used anti-diabetic drug, metformin, on mitochondrial function in STB from lean, obese, and GDM women in vitro and report several novel findings. Metformin treatment significantly decreased basal, ATP-linked, and maximal mitochondrial respiration as well as spare respiratory capacity (maximal minus basal) of STB mitochondria in a concentration-dependent manner, regardless of patient type, but only at concentrations above 0.1 mM. Metformin at concentrations (approximately 0.01 mM) found in maternal plasma of treated women [31,32,33,34,35] did not improve basal (unstressed state) respiration, ATP-linked respiration, maximal respiration, or spare capacity, but neither did it disrupt these functions [33,34]. As higher local metformin concentrations may be found due to accumulation in the mitochondria [35], further exploration of the relationship between circulating plasma and placental tissue mitochondrial metformin concentrations is warranted. Interestingly, a recently reported study also showed inhibition of basal, ATP-linked, and maximal respiration in trophoblast but at slightly lower concentrations (0.01–0.1 mM) [30]. No improvement in respiration was observed with metformin.

Metformin appears to impact STB in a sexually dimorphic manner, increasing spare capacity in female STB from A2GDM pregnancies, albeit at concentrations greater than those found in maternal plasma, but drastically increasing proton leak and uncoupled respiration in STB from males, especially in A2GDM pregnancies. Mitochondrial respiratory spare capacity is the ability of a cell to escalate oxidative phosphorylation in response to increased energy demand [43]. This may indicate that STB from female placentas from A2GDM women can better respond to demand for changes in energy requirements than males to increase their chances of survival in an adverse environment.

Metformin may benefit STB via affecting increased proton leak: this response observed in male STB following metformin treatment may be due to elevated ROS and act as a protective mechanism in cells against oxidative damage [44]. Male STB from A2GDM women may use this mechanism to limit damage to mitochondrial function and the cell. A recent publication by Ionică et al. demonstrated a reduction in ROS in cardiac tissue isolated from a rat diabetic model following metformin treatment, further strengthening this theory [45]. Elevated extracellular acidification would be expected in these cells; however, surprisingly, acidification was not significant at the metformin concentrations where proton leak data was most significant. One explanation could potentially lie in the fuel flexibility of the placenta, the ability to switch between the use of different classes of substrates. Non-glucose substrates result in more cellular acidification: the fatty acid palmitate can cause 60–100% of acidification, whereas glucose only accounts for 34% [31]. Male STB from A2GDM women are the least fuel-flexible, using primarily glucose rather than fatty acids to generate ATP, masking the acidification effect caused by proton leak [5]. A lack of fuel flexibility has also been observed as a programming consequence in mouse offspring following maternal metformin treatment [46]. Further explaining masking is another potential mechanism: metformin affects AMPK signaling. The increased AMP:ATP ratio caused by proton leak activates AMPK, which regulates several processes, including mTOR signaling and the phosphorylation of proteins involved in reducing inflammation and acidification [36,47].

Overall, metformin produces little glycolytic response in STB, despite the inhibitory effect on oxidative phosphorylation at 1 mM metformin and above. Metformin concentrations above 0.01 mM increased glycolysis but only in STB from lean women. In contrast, increased glycolytic capacity, in response to energy demand, occurred in the therapeutic range seen with metformin treatment exclusively in female STB from lean women, giving no beneficial effect to STB from pregnancies challenged by obesity or GDM. While metformin causes increased non-glycolytic acidification arising from lactate formation or glycogenolysis in STB from obese or A2GDM women, this occurs above the reported plasma concentration following treatment, so any in vivo effect would be dependent on metformin accumulation in mitochondria.

Metformin concentrations in the therapeutic range reduced mitochondrial superoxide release in STB from female placentas of lean and obese women. In obese women, this may be partly due to reduced mitochondrial content compared to sex-matched groups and matches with the reduction in mitochondrial respiration parameters in these groups. We observed no change in STB from A2GDM women regardless of fetal sex in response to increasing metformin. However, at 0.1 mM metformin, female STB of all groups had reduced superoxide release versus controls. This may be due to cellular senescence reducing activity rather than apoptosis. MitoTracker identifies intact mitochondria, including those in senescent cells, while MitoSox identifies mitochondria actively generating superoxide [48]. It is also possible that more mitochondria were formed in STB as seen in endometrial tumor cells in vitro, where metformin increased mitochondrial biogenesis while impairing mitochondrial function [49]. In contrast, lean male trophoblast mitochondria produce higher superoxide concentrations compared to other groups in the therapeutic range of treatment. Metformin may be deleterious to non-A2GDM STB, so a confirmed diagnosis is critical before considering treatment. Previous research indicated that STB mitochondria from A2GDM women were swollen or destroyed [50]. However, our study shows that viable mitochondrial content remained relatively unchanged with metformin treatment. Therefore, metformin may help stabilize A2GDM STB mitochondrial function by maintaining the quantity present, even when A2GDM pathology or metformin treatment impact function.

A major strength of this study was the use of primary human STB rather than cell lines and non-human models; furthermore, they were derived from cesarean delivery, which eliminates the effect of oxidative stress induced during labor. Animal studies also produced mixed results regarding metformin treatment efficacy. In mouse studies, the positive effects of metformin on GDM pregnancies include protecting neural cells against apoptosis and neural tube defects and reducing inflammatory reactions caused by angiotensin II, lipopolysaccharides, and cytokines [29]. However, metformin exposure during murine pregnancy also resulted in obese offspring in mice, with males having impaired glucose tolerance when given high-fat diets [46]. Given this, there is a concern for long-term fetal programming of metabolic syndromes following metformin treatment, amplified as we have shown that metformin may impact the placenta and, thereby, fetal development.

Further strengths included comparing effects in male and female placentas, knowing that many aspects of placental function are sexually dimorphic, and a comparison of effects in trophoblast from lean, obese, and type A2GDM pregnancies where we have previously demonstrated varying degrees of mitochondrial dysfunction related to oxidative stress which metformin may impact. Human placenta samples were carefully selected from our repository for each group to exclude confounding factors, including asthma, preeclampsia, and multiple gestation. A2GDM patients received insulin as a treatment but were metformin naive. Ethnicity is also a future factor to consider in pregnancy outcomes: maternal obesity has been associated with placental inflammation in Black women [51,52], and our experimental population was primarily Caucasian.

## 5. Conclusions

In conclusion, our data suggest that the effect of metformin varies based on maternal condition and fetal sex in vitro, with limited signs of improvement in STB from A2GDM women but potential harm in STB from lean women, particularly when the fetus is male. However, the majority of effects following metformin treatment in vitro were observed at concentrations exceeding those reached in maternal plasma in pregnant women treated with metformin to control their GDM. While it is reassuring that the deleterious effects of metformin on trophoblast respiration occur at concentrations exceeding those reported in maternal plasma in vivo, higher mitochondrial amounts may be reached via accumulation, which could lead to adverse effects and a compensatory glycolytic response, as observed in our assays at supraphysiologic metformin concentrations. Our findings support that the immediate and long-term developmental impact of metformin treatment during pregnancy must be carefully considered regarding the efficacy of metformin versus its risks.

## Figures and Tables

**Table 1:** Patient Characteristics. Mean ± SD. * *p* < 0.05 vs. Lean, ^#^ *p* < 0.05 vs. A2GDM of corresponding sex.

Maternal Group	Fetal Sex	Maternal Age (Years)	Gest. Age (Weeks)	BMI (kg/m^2^)	Fetal Weight (g)	Placental Weight (g)	Fetal/Placental Ratio
Lean	Male *n* = 5	39.5 ± 4.8	38.7 ± 1.0	21.7 ± 1.5	3546 ± 356	482 ± 60	7.3 ± 0.1
	Female *n* = 4	32.5 ± 3.3	38.3 ± 0.9	23.3 ± 1.2	3051 ± 471	470 ± 72	6.5 ± 0.2
Obese	Male *n* = 4	29.6 ± 8.1	39.3 ± 0.3	39.7 ± 10.9 *	3435 ± 982	541 ± 138	6.4 ± 0.3 *
	Female *n* = 5	31.5 ± 3.7	38.9 ± 0.6	36.5 ± 5.7 *	3642 ± 351	598 ± 87	6.1 ± 0.2 ^#^
A2GDM	Male *n* = 5	33.0 ± 3.2	38.3 ± 1.1	40.4 ± 6.7 *	3786 ± 516	550 ± 183	6.9 ± 0.2 *
	Female *n* = 5	33.2 ± 5.8	37.9 ± 1.4	38.4 ± 4.3 *	3836 ± 751	566 ± 127	6.8 ± 0.2

## Data Availability

Data is contained within the article.
